# Identifying the obstacles facing emergency nurses regarding treating CTAS1 and CTAS2 in Saudi Arabia

**DOI:** 10.1186/s12873-024-01044-4

**Published:** 2024-07-18

**Authors:** Rawan AlZahrani, Abdulellah Al Thobaity, Manal Saleh Moustafa Saleh

**Affiliations:** 1 King Faisal Medical Complex, Ministry of Health, Taif, Saudi Arabia; 2 Department of Medical Surgical Nursing, College of Nursing at Taif University, Taif, Saudi Arabia; 3 Nursing Department, College of Applied Medical Science, Shaqra University, Shaqra, Saudi Arabia; 4 Nursing Administration, Faculty of Nursing, Zagazig University, Zagazig, Alsharqia Egypt

**Keywords:** Triage, Emergency nurses, Emergency obstacles, Emergency challenges, Saudi Arabia

## Abstract

**Background:**

Emergency nurses play a pivotal role in delivering efficient emergency healthcare, yet they often encounter numerous challenges, especially while managing life-threatening cases, impacting both their well-being and patient satisfaction. This study seeks to identify the prevalent challenges faced by these nurses in Saudi hospitals when handling Canadian Triage and Acuity Scale (CTAS1 and CTAS2) cases, with the aim of mitigating or managing these issues in the future.

**Methods:**

This study incorporated a mixed-method approach to identify obstacles in Emergency Department (ED) nursing treatment of CTAS1 and CTAS2 cases in two major Saudi Arabian hospitals. The research began with qualitative focus group interviews with expert ED nurses, followed by a quantitative survey to measure and explore relationships among the qualitative findings. Data analysis leveraged qualitative thematic analysis and principal component analysis, ensuring rigorous examination and validation of data to drive meaningful conclusions.

**Findings:**

From expert interviews, key challenges for emergency nurses were identified, including resource management, communication, training compliance, and psychological factors. A survey of 172 nurses further distilled these into five major issues: patient care management, handling critical cases, administration support, patient care delay, and stress from patients’ families.

**Conclusion:**

Through a mixed-method approach, this study pinpoints five pivotal challenges confronting emergency nurses in Saudi hospitals. These encompass difficulties in patient care management, the psychological toll of handling critical cases, inadequate administrative support, delays due to extended patient stays, and the stress induced by the presence of patients’ families, all of which significantly impede emergency department efficiency and compromise nurse well-being.

## Background

Emergency departments (EDs) are crowded areas with complicated communication regions and a high rate of job interruptions and diversions [1]. ED is a place for urgent diagnoses and interventions for different ages and health conditions [2]. It is where high-quality and timely patient care is delivered, starting with sorting and organizing the healthcare flow provided to the patients when they enter the ED gates. The term “triage” is derived from the French word *trier*, which means sorting and arranging. Triaging is a system used in many areas of the health field to ensure patient safety by classifying patients according to the severity of their health problems. Then, they are prioritized for healthcare in a stable treatment area within a specific time frame. Triaging differs from one country to another, depending on the type of triaging system they follow. Many triaging systems were created, such as the simple triage and rapid treatment system, the Canadian Triage and Acuity Scale (CTAS), the Chinese four-level and three-district triage standard, the Australasian Triage Scale, and the Manchester Triage System [3, 4].

In Saudi Arabia, many hospitals use the Canadian Triage and Acuity Scale (CTAS) as a reliable method to categorize patients in the emergency department (ED) based on their needs [5]. CTAS has five levels, ranging from the most urgent (CTAS 1) to the least urgent (CTAS 5) [6, 7]. These levels cover different situations, including resuscitation, emergent care, urgent cases, less urgent cases, and non-urgent cases. CTAS 1 patients require immediate life-saving interventions due to critical conditions like heart attacks or severe breathing problems. They must be seen and treated promptly to prevent worsening or death [6, 8]. CTAS 2 patients have serious but not critical conditions needing quick assessment and care, typically within 15 min [6, 8]. Examples include chest pain, heavy bleeding, or high-risk pregnancy complications. Although not as urgent as CTAS 1, CTAS 2 patients still need timely attention to avoid further issues. At each level, the ED nurse and physician have limited time to assess the situation, though the issue is the lack of national fixed unified standardized criteria for triaging patients in Saudi hospitals [5]. The one who performs this complicated process as well as other ED processes is the emergency nurse.

Emergency nurses play an essential role in the success of providing care and treatment to trauma victims. Before a definitive diagnosis is determined and while patients are awaiting a doctor, nurses may begin basic therapy [9]. Emergency nurses deal with unpredictable loads of people, patients, and their families with different clinical problems and who need care within a compressed time frame, along with frequent fluctuations in patients’ acuity of conditions. The educational criteria for emergency nurses in Australia are among the highest in the world [10]. In the Kingdom of Saudi Arabia (KSA), emergency nursing is one of the postgraduate professional training programs offered under the leadership of the Saudi Commission for Health Specialties as an improvement step to the national Vision 2030 [11]. Emergency nurses work in an area that requires a high level of professionalism and attention to meet the requirements of patient safety and provide care with good quality. This task is not easy, particularly in an area that faces national and international obstacles.

WHO and the World Bank estimated 9 million nurses and midwives were needed worldwide in 2014. By 2030, they projected the figure would drop to 7.6 million [12]. Specialty fields, such as emergency nursing, will not be exempt from this severe shortage [13]. Many studies found that emergency nurses experience workplace violence (WPV) at a high rate worldwide. In a study published in 2019, 89.9% of 385 emergency nurses in 13 Chinese general hospitals experienced WPV in 2018 [14]. Furthermore, a review study of violent incidents rates conducted in 18 different countries showed that 21–82% of verbal violence and 13–79% of physical truculence were reported by ED professionals [15]. Moreover, a study conducted in Saudi Arabia in 2021 found that 73.7% of 849 emergency nurses in the last two years express WPV in public health hospitals [16]. A quantitative study conducted in three different university hospitals in France aimed to measure the prevalence of burnout symptoms among ED professionals. The study found that of 379 participants, 15.8% and 29.6% reported that they experience burnout syndrome [17]. In southern Saudi Arabia, a cross-sectional study was conducted in hospitals to assess burnout among emergency staff; among the 282 respondents, 66.5% were nurses, of whom 16.3% showed prevalence of burnout [18]. Emergency nurses struggle daily with several aspects affecting their job satisfaction, intention of turnover, and negative physical or emotional impact. Abundant evidence-based research is conducted regarding the barriers faced by emergency nurses. However, each research discusses one obstacle as a topic isolated from other obstacles.

Based on available evidence from published studies, further evidence-based research is necessary to collect, explore, and distinguish the most important challenges faced by emergency nurses, especially when dealing with CTAS1 and CTAS2 cases. Accordingly, this study aimed to explore the most significant challenges faced by emergency nurses when dealing with CTAS1 and CTAS2 cases in Saudi hospitals to avoid or manage them in the future.

## Methods

This study employed a mixed-method approach with sequential analysis, beginning with a qualitative phase followed by a quantitative phase based on the initial findings. The first phase involved focus group interviews with expert Emergency Department (ED) nurses from King Abdul-Aziz Specialist Hospital and King Faisal Medical Complex. This phase aimed to explore and identify the obstacles in ED nursing regarding the treatment of CTAS1 and CTAS2 cases. The second phase involved a quantitative survey to quantify the obstacles that identified in the first phase. The study was conducted in two major hospitals in Taif, Saudi Arabia: King Faisal Medical Complex Hospital, which has a capacity of over 500 beds and a large ED with around 50 beds, and King Abdul-Aziz Specialist Hospital, with a bed capacity of approximately 500 beds and an ED consisting of about 30 beds. In the initial phase, from July to September 2022, two distinct focus group interviews were conducted, each comprising four participants from a different hospital. This approach was chosen to manage logistical constraints and to highlight any potential variances between the practices of the two hospitals.

Each focus group was conducted by a single facilitator, a registered nurse with over 10 years of experience in emergency care and expertise in qualitative research methods. This facilitator had extensive training in conducting focus groups and in-depth interviews, which ensured that the discussions were guided effectively and remained focused on the research objectives. Importantly, the facilitator had no prior relationship with the participants, which helped to minimize bias and encouraged open, honest dialogue. The discussions were facilitated using a guide that included open-ended questions, as outlined in Table 1. This guide was designed to elicit detailed responses about the challenges faced in managing critical cases, such as CTAS1 and CTAS2 patients, including issues related to supply availability and administrative processes. The open-ended nature of the questions allowed participants to express their views freely and provided rich, nuanced data. Each focus group session lasted between 30 and 45 min and was audio-recorded with the informed consent of the participants. This ensured an accurate record of the discussions. In addition to the recordings, the facilitator took field notes to capture non-verbal cues and contextual details that might not be evident from the audio recordings alone. These notes provided additional depth to the analysis.

**Table 1:** Focus group discussion questions

Q1. What are the negative points about working in the ED, particularly in CTAS1 and CTAS2?
Q2. What are the challenges faced by nurses in these two areas in the ED?
Q3. What about the availability of supply in CTAS1 and CTAS2?
Q4. Do you agree that dealing with emergency cases daily can affect nurses? How?
Q5. What can be done to reduce the challenges for nurses in the ED?

Data saturation was achieved when no new themes or insights emerged from the second focus group, indicating that the data collected were sufficient to address the research questions. The audio recordings were transcribed verbatim by a professional transcription service to ensure accuracy. Although the transcripts were not returned to participants for verification, the researchers shared key themes and preliminary findings with the participants to confirm the accuracy of the interpretations and to obtain their feedback. The data were analyzed using qualitative thematic analysis. Two researchers independently coded the transcripts to identify key themes and patterns. This independent coding process helped to enhance the reliability of the findings. Any discrepancies between the researchers’ codes were resolved through discussion and consensus, ensuring that the final themes accurately reflected the data. NVivo software was used to support the analysis and manage the data systematically, providing a robust framework for organizing and retrieving coded data.

The study adhered to the Consolidated Criteria for Reporting Qualitative Research (COREQ) checklist, ensuring that all relevant methodological details were transparently reported. This adherence to COREQ standards enhanced the credibility and rigor of the study, providing a clear and comprehensive account of the research process.

The second phase was a cross-sectional study conducted through an open-link survey using SurveyMonkey (surveymonkey.com). The questionnaire was developed based on the results from the first phase and comprised two parts: participants’ demographic data and 28 items related to the study topic, as detailed in the Results section. Data collection occurred from November 2022 to December 2022. The survey was promoted via official hospital communication channels to reach our target respondents—all staff nurses working in the ED. To ensure a comprehensive and effective advertising strategy, researchers visited the hospitals personally and coordinated with the education and research units. Before the official distribution, the questionnaire was pilot-tested with a small group of nurses to evaluate its clarity and relevance. Feedback from this pilot test was invaluable, leading to minor adjustments in the questionnaire to enhance its effectiveness and reliability.

A 5-point Likert scale was used 1 = strongly disagree and 5 = strongly agree. Data used in this phase were collected from the same two large hospitals in Taif where the focused group data were taken. The inclusion criteria comprise all staff nurses working in the ED; the exclusion criteria are students and nursing interns who work in the ED. The expected population in both EDs is approximately 225; thus, the estimated number of participants is 169 (confidence level 99%; margin of error 5). A brief explanation is provided to them indicating that they have the right to withdraw from the survey with no risk or harm to them. The survey results were collected directly into the investigator’s account, and data were stored in the responsible investigator’s laptop for 5 years to maintain confidentiality. Descriptive statistics and principle component analysis (PCA) using SPSS (version 25) [19] were used for data analysis. PCA was conducted to validate and remove redundant data following the process of factor analysis adapted from: ***Exploratory factor analysis: A five-step guide for novices*** [20]. This process follows five steps: [1] data suitability based on the sample size and the Kaiser–Meyer–Olkin measure of sampling adequacy and Bartlett’s test of sphericity; [2] factor extraction methods using the PCA as not a priori knowledge regarding the research topic; [3] criteria for determining the factor extraction method based on the eigenvalue greater than 1 rule and explained variance more than 50%; [4] selection of a rotation method using varimax, which is the most commonly used method and maximizes high item loadings; and [5] interpretation by deleting items with cross-loadings and those with no loading to any factors and naming the factors according to the characteristics of the items.

## Results

The results of this study are presented sequentially as well as the method used.

### Approach 1: Qualitative phase

Following the interview conducted, we generated items for the purpose of developing a scale for the quantitative phase, as shown in Table 2. From these items, we identified four major themes: (1) Staffing and Resource Management, which encompasses challenges related to understaffing, shortages of supplies and equipment, and the availability of hospital beds for critical cases; (2) Communication and Information Sharing, highlighting the importance of clear and accurate communication between healthcare providers, patients, and their families; (3) Training, Education, and Compliance, covering the need for proper training and education for healthcare professionals working in the ED, as well as ensuring compliance with established procedures; and (4) Psychological, Emotional, and Workflow Factors, focusing on the emotional and psychological well-being of healthcare professionals and addressing workflow and process challenges in the ED.

**Table 2:** Items extracted from the interviews

1. Understaffing affects my duty hours in the ED
2. Staff shortage is a major problem in the ED
3. The presence of patients and their families during treating cases adds pressure on me.
4. I get stressed when patients’ families asked me questions that I don’t know how to answer.
5. The consultation process in ED delays the medical care for critical patients.
6. Assigning new staff in CTAS1 and CTAS2 may affect critical patient care.
7. Improper pre-hospital assessment, intervention, and transportation for critical patients affect patient care in CTAS1 and CTAS2.
8. Inadequate information about a referred patient is an issue in CTAS1 and CTAS2.
9. Receiving incorrect endorsement about a patient’s condition from pre-hospital health providers affects the receiving preparation in CTAS1 and CTAS2.
10. Doctors’ non-compliance with the procedures in the ED affects my performance in treating critical patients in CTAS1 and CTAS2.
11. Receiving multiple orders during code blue confuses nurses.
12. The presence of internship students and new doctors during code blue affects nurses’ monitoring and quality care.
13. The policies and protocols in my ED conflict.
14. Increases in patients’ ED length of stay (EDLOS) due to delayed doctor assessment are a major problem.
15. Shortage of supplies in the ED affects patient care.
16. Equipment in the ED is insufficient, affecting patient care.
17. The lack of coping mechanisms with critical cases affects my lifestyle.
18. Frequent dealings with critical cases affect my imagination and self-stability.
19. Frequent critical care negatively influences my self-care.
20. The lack of training and education in dealing with critical care patients in the ED
21. Improper hospital administration affects workflow in the ED.
22. Separation of hospital administration from emergency administration leads to changes in the workplace environment.
23. A lack of motivation and psychological support affects the performance of emergency nurses working in CTAS1 and CTAS2.
24. Repositioning patients between emergency areas is one of the most challenges faced by nurses in the ED.
25. The lack of hospital beds for critical cases forces emergency nurses to continue nursing care for an extended period.
26. How doctors deliver bad news regarding patient care in the ED affects the workplace environment.
27. The occurrence of deaths in the ED affects nursing psychology.
28. Any medical error, regardless of how small, that occurs in CTAS1 and CTAS2 affects the patient healthcare process.

### Approach 2: Quantitative phase

In the second phase, 172 nurses responded to and returned the questionnaire, representing a response rate of 76.5%. The majority of the participants were female (82.6%), whereas the response rate of males was 17.4%. In terms of age, the majority were 29–38 years (53.5%), followed by 18–28 years (27.9%), 39–48 years (16.3%), and 49–58 years (2%). Most of the participants were Saudi nationals (88.4%). Regarding experience in emergency care settings, 54.1%, 13.4%, 12.2%, 9.9%, and 8.7% had 1–5 years, 7 months–1 year, 6–10 years, 11–15 years, and less than 6 months of experience, respectively. In terms of training and education courses, the majority of the participants have Conscious Sedation Course (43.6%). Others have Triaging Course (19.8%), ATLS Course (10.5%), and Fundamental Critical Care Support (4.7%). Then, the remaining participants (21.5%) have other training and education courses. In terms of emergency qualification, 57.0% have BLS, 27.9% had ATLS, and only 7.0% had PALS. Approximately 20% had other forms of education and training (critical care ED, ATCN, BLE, NRP, CRRT). Table 3 presents additional details.

**Table 3:** Demographic data

Group	Subgroup	Frequency	Percentage
Gender	Male Female Total	30 142 172	17.4 82.6 100
Age in years	18–28 29–38 39–48 49–58 Total	48 92 28 4 172	27.9 53.5 16.3 2.3 100
Nationality	Saudi Non-Saudi Total	152 20 172	88.4 11.6 100
Years of ED experience	Less than 6 months 7 months–1 year 1–5 years 6–10 years 11–15 years More than 15 years Total	15 23 93 21 17 3 172	8.7 13.4 54.1 12.2 9.9 1.7 100
Emergency care qualification	BLS ACLS PALS Others Total	98 48 12 14 172	57.0 27.9 7.0% 8.2 100.1
Training and education course	Conscious Sedation Course ATLS Course Triaging Course Fundamental Critical Care Support ) Others Total	75 18 34 8 37 172	43.6 10.5 19.8 4.7 21.5 100.1

The findings of PCA indicate that a sample size of more than 100 is suitable for factor analysis; the Kaiser–Meyer–Olkin measure of sampling adequacy is 0.82; and Bartlett’s test of sphericity approx. chi-square is 2082.24, p < 0.001. Based on the eigenvalues and total variance explained, five factors can be extracted from the data as they have eigenvalues greater than 1, with 65% total variance explained. Based on the criteria of factor loadings of more than 0.50, five items were deleted due to no loading or cross-loading to two factors or more. Thus, 23 items remain from the original 28 items. With approximately 65% total variances, five factors were extracted, and each factor scored an eigenvalue greater than 1. Thus, this study focuses on exploring the five factors as the most important obstacles and challenges faced by emergency nurses in treating CTAS1 and CTAS2 cases in Saudi hospitals to avoid or manage them in the future (Table 4). The first factor was patient care management, with nine items related to healthcare providers’ attitudes affecting patient care. Patient care management has factor loadings from 0.77 to 0.50, with two items having the same loading of 0.70 (“Any medical error, regardless of how small, that occurs in CTAS1 and CTAS2 affects the patient’ healthcare process” and “Receiving incorrect endorsement about a patient’s condition from pre-hospital health providers affects the receiving preparation in CTAS1 and CTAS2”). The highest loading pertains to “Inadequate information about a referred patient is an issue in CTAS1 and CTAS2,” with M = 3.87, SD = 0.92, 8.44 eigenvalues, and 36.70% variance. The second factor was dealing with critical cases involving four items, with 2.19 eigenvalues and 9.53% variance and loadings ranging from 0.81 to 0.61. In terms of the strongest to the weakest mean values, “The occurrence of deaths in the ED affects nursing psychology” has M = 3.70 and SD = 0.98; “The lack of coping mechanisms with critical cases affects my lifestyle” has M = 3.58 and SD = 0.96; “Frequent dealings with critical cases affect my imagination and self-stability” has M = 3.43 and SD = 1.04; and “Frequent critical care negatively influences my self-care” has M = 3.05 and SD = 1.11. The third factor was hospital administration support comprising five items with loadings from 0.717 to 0.547, 2.01 eigenvalue, and 8.70% variance, one items higher than 0.70 was : Improper hospital administration affects workflow in the ED (0.717). The fourth factor was a delay in patient care involving three items with loading ranged from 0.870 to 0.613. The items with a strong loading higher than 0.80 were “Shortage of supplies in the ED affects patient care” with M = 3.57 and SD = 1.13 and “Increases in patients’ ED length of stay (EDLOS) due delayed doctor assessment are a major problem,” with M = 3.54 and SD = 1.40. Lastly, the fifth factor has 2 items loaded alone (from 0.80 to 0.72). Both items focused on how the presence of patients’ families stresses emergency nurses.

**Table 4:** Principal component analysis results

Items	Factors							
	1	2	3	4	5	h	M	SD
Inadequate information about a referred patient is an issue in CTAS1 and CTAS2	0.77					0.63	3.87	0.92
Improper pre-hospital assessment, intervention, and transportation for critical patients affect patient care in CTAS1 and CTAS2	0.76					0.75	3.98	0.96
Any medical error, regardless of how small, that occurs in CTAS1 and CTAS2 affects the patient’s healthcare process	0.70					0.63	4.12	0.94
Receiving incorrect endorsement about a patient’s condition from pre-hospital health providers affects the receiving preparation in CTAS1 and CTAS2	0.70					0.73	3.97	0.86
Doctors’ non-compliance with the procedures in the ED affects my performance in treating critical patients in CTAS1 and CTAS2	0.68					0.63	3.92	0.91
A lack of motivation and psychological support affects the performance of emergency nurses working in CTAS1 and CTAS2	0.64					0.61	3.91	0.93
Assigning new staff in CTAS1 and CTAS2 may affect critical patient care	0.60					0.52	3.86	0.94
How doctors deliver bad news regarding patient care in ED affects the workplace environment	0.57					0.61	3.68	0.93
Receiving multiple orders during code blue confuses nurses	0.50					0.50	3.88	0.94
Frequent dealings with critical cases affect my imagination and self-stability		0.81				0.72	3.43	1.04
Frequent critical care negatively influences my self-care		0.77				0.73	3.05	1.11
The occurrence of deaths in the ED affects nursing psychology		0.68				0.65	3.70	0.98
The lack of coping mechanisms with critical cases affects my lifestyle		0.61				0.55	3.58	0.96
Improper hospital administration affects workflow in the ED			0.717			0.68	3.95	0.87
The presence of internship students and new doctors during code blue affects nurses’ monitoring and quality of care			0.620			0.50	3.70	0.94
Equipment in the ED is insufficient, affecting patient care			0.619			0.60	3.81	0.99
Separation of hospital administration from emergency administration leads to changes in the workplace environment			0.548			0.50	3.80	0.78
The lack of training and education in dealing with critical care patients in the ED			0.547			0.52	3.71	0.96
Increases in patients’ EDLOS due to delayed doctor assessment are a major problem				0.870		0.76	3.54	1.40
Shortage of supplies in the ED affects patient care				0.849		0.78	3.57	1.13
The consultation process in ED delays the medical care for critical patients				0 0.613		0.60	3.71	0.95
I get stressed when patients’ families asked me questions that I don’t know how to answer					0.80	0.79	3.35	1.06
The presence of patients’ families when treating cases adds pressure on me					0.72	065	3.81	0.99
Eigenvalues	8.44	2.19	2.01	1.21	1.01			
% Variance	36.70	9.53	8.70	5.27	4.39			

## Discussion

This study highlights five major challenges faced by emergency nurses in Saudi hospitals when handling Canadian Triage and Acuity Scale (CTAS1 and CTAS2) cases: patient care management, handling critical cases, hospital administration support, patient care delays, and dealing with patients’ families. It emphasizes the crucial role of nursing care in ensuring patient safety, quality, and satisfaction [21]. One key finding is that patient care management challenges significantly impact healthcare providers’ attitudes, which in turn affect healthcare processes and outcomes, particularly in critical cases. Issues such as errors in triage and inadequate information from pre-hospital health providers disrupt patient care. Medical errors, a leading cause of patient deaths in the US, underscore the need for error management and patient safety in emergency departments, where risks are high [22–26].

The study also highlights the importance of effective communication and thorough information exchange during referrals, especially for referred patients in urgent categories. It is reported that a significant number of emergency nurses have made medical errors, emphasizing the need for reduced nurse workloads and improved conditions to mitigate such errors [27, 28]. International standards advocate for direct communication and complete information transfer during inter-hospital transfers, with ineffective interactions, handoffs, and IT shortcomings impacting patient transfers [29]. Effective nurse-doctor communication in the ED is vital for collaboration [30]. Communication barriers between emergency nurses and other team members can obstruct care delivery, with communication errors during handovers posing significant risks [31–33]. A UK survey from 2005 linked over 80% of critical transfer incidents to inadequate training and equipment [34]. Patient satisfaction and safety, as measures of ED care quality, support the importance of managing patient care effectively [35, 36].

The study identifies the impact of dealing with critical cases on healthcare providers, with significant statistical measures (eigenvalues of 2.19, 9.53% variance) and strong item loadings (0.81 to 0.61). Frequent encounters with death in the ED take a toll on nurses’ psychological well-being, affecting their lifestyle, imagination, self-stability, and self-care, with varying degrees of impact on their coping mechanisms. The highest mean value within this factor suggests a heavy emotional burden on emergency nurses, highlighting the need for psychological support and coping strategies within the ED. The study emphasizes the adverse effects on personal lifestyle and mental health, underscoring the necessity for stress management education and resilience training. It also suggests regular mental health assessments and access to psychological counseling to prevent burnout, alongside promoting self-care practices among healthcare workers.

These findings align with research indicating that direct exposure to death, workplace violence, and traumatic events can lead to post-traumatic stress disorder in emergency nurses. Strategies such as psychological support, job training, and optimized shift schedules are recommended to safeguard nurses’ physical and mental health [37]. Similarly, a study in Thailand highlights the importance of hospital management strategies, including support from co-workers and supervisors, to mitigate psychological effects and occupational stress in ED nurses [38]. The literature consistently suggests that without adequate emotional recovery interventions, nurses dealing with critical cases may experience post-traumatic stress, emotional exhaustion, and compassion fatigue [39, 40].

The study also emphasizes the critical role of hospital administration support for the smooth operation of the Emergency Department (ED). Inadequate administrative support can disrupt ED operations, leading to increased medical errors and patient dissatisfaction. It has been note that inadequate administrative support may elevate stress levels among healthcare providers, negatively affecting their health and care delivery, potentially increasing turnover rates [41, 42]. Fostering a close relationship between administration and nursing staff enhances motivation and underscores the importance of considering the psychological well-being of nurses [43]. These findings suggest that supportive and efficient hospital administration is essential for a positive work environment, improved ED workflow, and ultimately better patient outcomes.

The study also highlights the issue of delay in patient care, which includes supply shortages and increased emergency department length of stay (EDLOS) due to delayed doctor assessments. Supply shortages can hamper timely care delivery and exacerbate patient conditions, emphasizing the importance of resource management and efficient inventory systems [44, 45]. A study highlighted the correlation between supply availability and patient care quality, indicating the need for operational adjustments and emergency management protocols to prepare EDs for high patient volumes [46, 47]. Additionally, delays in assessments lead to extended wait times, potentially deteriorating patient conditions and reducing satisfaction. Measures such as optimizing workforce levels and changing triage processes can significantly reduce waiting times [48, 49]. Overall, these factors highlight the challenges faced by emergency nurses in patient care management. Addressing these challenges through effective communication, psychological support, supportive hospital administration, and efficient resource management can contribute to improved patient outcomes, satisfaction, and the well-being of healthcare providers.

## Conclusions

In conclusion, this study underscores the significant challenges faced by emergency nurses in patient care management and highlights the importance of addressing these challenges for improved healthcare outcomes. The findings emphasize the need for effective communication, psychological support, supportive hospital administration, and efficient resource management. By addressing these factors, healthcare organizations can enhance the quality of emergency care, promote patient satisfaction, and prioritize the well-being of healthcare providers. It is crucial to recognize and mitigate the challenges faced by emergency nurses to ensure optimal patient outcomes and a positive work environment within the Emergency Department.

### Limitation

This study, despite providing important insights, is not without limitations. The research was conducted only in two major Saudi hospitals, which might limit the generalizability of findings to different geographical regions or types of hospitals. The reliance on self-reported data may have introduced biases such as recall or social desirability bias. Furthermore, the relatively small sample size of 172 nurses may further restrict the applicability of the findings across a broader context. Lastly, the study did not deeply delve into the root causes of these challenges or potential solutions, which could be addressed in future research for a more comprehensive understanding and for the development of targeted interventions.

## Data Availability

The datasets used and/or analysed during the current study are available from the corresponding author on reasonable request.

## References

[1] Amaniyan S, Faldaas BO, Logan PA, Vaismoradi M (2020). Learning from patient safety incidents in the emergency department: a systematic review. J Emerg Med.

[2] Ortíz-Barrios MA, Alfaro-Saíz J-J (2020). Methodological approaches to support process improvement in emergency departments: a systematic review. Int J Environ Res Public Health.

[3] Yancey CC, O’Rourke MC. Emergency department triage. 2020.

[4] Reay G, Smith-MacDonald L, Then KL, Hall M, Rankin JA (2020). Triage emergency nurse decision-making: incidental findings from a focus group study. Int Emerg Nurs.

[5] Alquraini M, Awad E, Hijazi R (2015). Reliability of Canadian emergency department triage and acuity scale (CTAS) in Saudi Arabia. Int J Emerg Med.

[6] Bullard MJ, Musgrave E, Warren D, Unger B, Skeldon T, Grierson R (2017). Revisions to the Canadian emergency department triage and acuity scale (CTAS) guidelines 2016. Can J Emerg Med.

[7] Viana J, Bragança R, Santos JV, Alves A, Santos A, Freitas A (2023). Validity of the Paediatric Canadian Triage Acuity Scale in a Tertiary Hospital: an analysis of severity markers’ variability. J Med Syst.

[8] Lee JY, Oh SH, Peck EH, Lee JM, Park KN, Kim SH (2011). The validity of the Canadian triage and acuity scale in predicting resource utilization and the need for immediate life-saving interventions in elderly emergency department patients. Scand J Trauma Resusc Emerg Med.

[9] Awwad K, Ng YG, Lee K, Lim PY, Rawajbeh B (2021). Advanced Trauma Life Support/Advanced Trauma Care for nurses: a systematic review concerning the knowledge and skills of emergency nurse related to trauma triage in a community. Int Emerg Nurs.

[10] Cameron P, Jelinek G, Kelly A-M, Murray L, Brown A. Textbook of adult emergency medicine. New York. 2015.

[11] Aljohani KAS. Nursing education in Saudi Arabia: history and development. Cureus. 2020;12(4). 10.7759/cureus.7874 PMC7255546 32489728

[12] Drennan VM, Ross F (2019). Global nurse shortages: the facts, the impact and action for change. Br Med Bull.

[13] Gorman VL-A (2019). Future emergency nursing workforce: what the evidence is telling us. J Emerg Nurs.

[14] Li N, Zhang L, Xiao G, Chen J, Lu Q (2019). The relationship between workplace violence, job satisfaction and turnover intention in emergency nurses. Int Emerg Nurs.

[15] Ashton RA, Morris L, Smith I (2018). A qualitative meta-synthesis of emergency department staff experiences of violence and aggression. Int Emerg Nurs.

[16] Alsharari AF, Abu-Snieneh HM, Abuadas FH, Elsabagh NE, Althobaity A, Alshammari FF (2022). Workplace violence towards emergency nurses: a cross-sectional multicenter study. Australasian Emerg care.

[17] Moukarzel A, Michelet P, Durand A-C, Sebbane M, Bourgeois S, Markarian T et al. Burnout syndrome among emergency department staff: prevalence and associated factors. BioMed Res Int. 2019;2019. 10.1155/2019/6462472 PMC6360614 30800675

[18] Alqahtani AM, Awadalla NJ, Alsaleem SA, Alsamghan AS, Alsaleem MA. Burnout syndrome among emergency physicians and nurses in Abha and Khamis Mushait cities, Aseer region, southwestern Saudi Arabia. Scientific World J. 2019;2019. 10.1155/2019/4515972 PMC6398028 30906233

[19] Corportation I (2017). IBM SPSS statistics for windows (version 25.0 Armonk).

[20] Williams B, Onsman A, Brown T (2010). Exploratory factor analysis: a five-step guide for novices. Australasian J Paramedicine.

[21] Oldland E, Botti M, Hutchinson AM, Redley B (2020). A framework of nurses’ responsibilities for quality healthcare—exploration of content validity. Collegian.

[22] Alnaser HNN, Al Yami DSM, Alrashah KAH, Al Zamanan AMA, Alnaser KNN, Alyami NAM et al. Prevention and reduction of medical errors: a narrative review. Annals Clin Anal Med. 2023;10(1).

[23] Khaleghi P, Akbari H, Alavi NM, Kashani MM, Batooli Z (2022). Identification and analysis of human errors in emergency department nurses using SHERPA method. Int Emerg Nurs.

[24] Robertson JJ, Long B (2018). Suffering in silence: medical error and its impact on health care providers. J Emerg Med.

[25] Freund Y, Goulet H, Bokobza J, Ghanem A, Carreira S, Madec D (2013). Factors associated with adverse events resulting from medical errors in the emergency department: two work better than one. J Emerg Med.

[26] Australian M. NZCoAACoE. Minimum standards for transport of the critically ill. Policy Doc. 1992:P23.

[27] Wang J, Mu K, Gong Y, Wu J, Chen Z, Jiang N (2023). Occurrence of self-perceived medical errors and its related influencing factors among emergency department nurses. J Clin Nurs.

[28] Kaur AP, Levinson AT, Monteiro JFG, Carino GP (2019). The impact of errors on healthcare professionals in the critical care setting. J Crit Care.

[29] Abraham J, Reddy MC (2010). Challenges to inter-departmental coordination of patient transfers: a workflow perspective. Int J Med Informatics.

[30] Saleh Moustafa Saleh M, Elsaid Elsabahy H, Abdel-Latif Abdel-Sattar S, Mohammed Atiea Mohammed K (2019). Developing and validating a strategic plan for total quality management in nursing. Egypt J Health Care.

[31] Al Badawi AK, Nashwan AJ (2024). The future of prehospital emergency care: embracing AI applications in ambulance services. Int Emerg Nurs.

[32] Sanjuan-Quiles Á, del Pilar Hernández-Ramón M, Juliá-Sanchis R, García-Aracil N, Castejón-de la Encina ME, Perpiñá-Galvañ J (2019). Handover of patients from prehospital emergency services to emergency departments: a qualitative analysis based on experiences of nurses. J Nurs Care Qual.

[33] Al Thobaity A (2020). An exploration of barriers to patients’ safety from the perspective of emergency nurses. Saudi J Health Sci.

[34] Stevenson A, Fiddler C, Craig M, Gray A (2005). Emergency department organisation of critical care transfers in the UK. Emerg Med J.

[35] Trout A, Magnusson AR, Hedges JR (2000). Patient satisfaction investigations and the emergency department: what does the literature say?. Acad Emerg Med.

[36] Nekoei-Moghadam M, Raadabadi M, Heidarijamebozorgi M (2020). Patient safety culture in university hospital’s emergency departments: a case study. Int J Health Plann Manag.

[37] Jiaru J, Yanxue Z, Wennv H. Incidence of stress among emergency nurses: a systematic review and meta-analysis. Medicine. 2023;102(4). 10.1097/MD.0000000000031963 PMC9875949 36705357

[38] Yuwanich N, Sandmark H, Akhavan S (2016). Emergency department nurses’ experiences of occupational stress: a qualitative study from a public hospital in Bangkok, Thailand. Work.

[39] Belayneh Z, Zegeye A, Tadesse E, Asrat B, Ayano G, Mekuriaw B (2021). Level of anxiety symptoms and its associated factors among nurses working in emergency and intensive care unit at public hospitals in Addis Ababa, Ethiopia. BMC Nurs.

[40] Sandler M (2018). Why are new graduate nurses leaving the profession in their first year of practice and how does this impact on ED nurse staffing? A rapid review of current literature and recommended reading. Can J Emerg Nurs.

[41] Jones JF. Strategies to increase Emergency Department patient Flow. Walden University; 2023.

[42] Hossny EK, Morsy SM, Ahmed AM, Saleh MSM, Alenezi A, Sorour MS (2022). Management of the COVID-19 pandemic: challenges, practices, and organizational support. BMC Nurs.

[43] Gao X, Jiang L, Hu Y, Li L, Hou L (2020). Nurses’ experiences regarding shift patterns in isolation wards during the COVID-19 pandemic in China: a qualitative study. J Clin Nurs.

[44] Abu Zwaida T, Pham C, Beauregard Y (2021). Optimization of inventory management to prevent drug shortages in the hospital supply chain. Appl Sci.

[45] Atcha P, Vlachos I, Kumar S. Inventory sharing in healthcare supply chains: systematic literature review and future research agenda. Int J Logistics Manage. 2023.

[46] Whiteside T, Kane E, Aljohani B, Alsamman M, Pourmand A (2020). Redesigning emergency department operations amidst a viral pandemic. Am J Emerg Med.

[47] Nadarajan GD, Omar E, Abella BS, Hoe PS, Do Shin S, Ma MH-M (2020). A conceptual framework for emergency department design in a pandemic. Scand J Trauma Resusc Emerg Med.

[48] Pryce A, Unwin M, Kinsman L, McCann D (2021). Delayed flow is a risk to patient safety: a mixed method analysis of emergency department patient flow. Int Emerg Nurs.

[49] Srinivas S, Nazareth RP, Shoriat Ullah M (2021). Modeling and analysis of business process reengineering strategies for improving emergency department efficiency. Simulation.

